# Physiological Stress Response to Sulfide Exposure of Freshwater Anaerobic Methanotrophic Archaea

**DOI:** 10.1021/acs.est.4c12489

**Published:** 2025-05-19

**Authors:** Maider J. Echeveste Medrano, Sarah Lee, Rob de Graaf, B. Conall Holohan, Irene Sánchez-Andrea, Mike S. M. Jetten, Cornelia U. Welte

**Affiliations:** † Department of Microbiology, Radboud Institute for Biological and Environmental Sciences (RIBES), 6029 Radboud University, Heyendaalseweg 135, 6525AJ Nijmegen, The Netherlands; ‡ Department of Environmental Sciences for Sustainability, 367407 IE University, C. Cardenal Zúñiga 12, 40003 Segovia, Castilla-Leon, Spain; § Laboratory of Microbiology, Wageningen University, Stippeneng 4, 6708WE Wageningen, The Netherlands

**Keywords:** ANME archaea, sulfide stress, sulfur oxidation, methanotrophy, methane

## Abstract

Freshwater wetlands and coastal sediments are becoming hotspots for the emission of the greenhouse gas methane. Eutrophication-induced deposition of organic matter leads to elevated methanogenesis and sulfate reduction, thereby increasing the concentrations of methane and toxic sulfide, respectively. However, the effects of sulfide stress on the anaerobic methanotrophic biofilter have not been well explored. Here, we show how an enrichment culture dominated by the freshwater anaerobic methane-oxidizing archaeon “*Candidatus* (*Ca*.) Methanoperedens” responds to short-term and long-term exposure to sulfide in a bioreactor. The methane-oxidizing activity decreased to 45% and 20% but partially recovered to 70% and 30% within 5 days after short- and long-term sulfide exposure, respectively. Metagenomics indicated that “*Ca*. Methanoperedens” remained dominant in the enrichment throughout the entire experiment. The first short-term sulfide pulse led to increased expression of genes encoding for sulfide detoxification by low abundant community members, whereas long-term exposure resulted in upregulation of “*Ca*. Methanoperedens” genes encoding sulfite reductases of group III (Dsr-LP). “*Ca*. Methanoperedens” consumed polyhydroxyalkanoates during long-term sulfide exposure, possibly to aid in stress adaptation. Together, these results provide a valuable baseline for understanding fundamental ecophysiological adaptations to methane cycling in sulfate- and nitrate-rich aquatic ecosystems.

## Graphical abstract

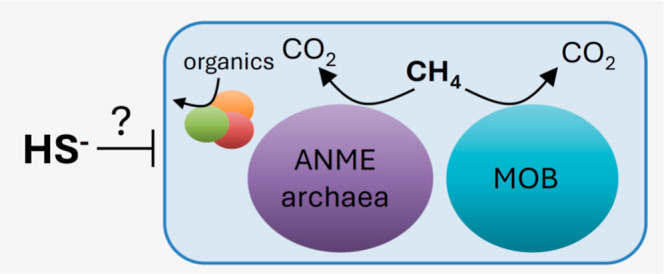

## Introduction

Methane is a potent greenhouse gas and is produced in anoxic environments by methanogenic archaea that use a limited number of substrates including H_2_/CO_2_, methanol, acetate, and dimethyl sulfide.^1^ A significant proportion of the methane produced is subsequently removed by anaerobic and aerobic methanotrophic microorganisms before reaching the atmosphere.^2^ The aerobic oxidation of methane is mediated by methane-oxidizing bacteria (MOB). Many MOBs have the genomic potential for (partial) denitrification and fermentation, occurring in the anoxic zones of lakes.^3^ Some reports also document the use of metal oxides by MOB.^4^ Furthermore, methanotrophs of the NC10 phylum or “*Candidatus* (*Ca*.) Methylomirabilis” can use nitrite as the electron acceptor and dismutate internally produced nitric oxide into oxygen and nitrogen gas, using the produced oxygen for the activation of methane via methane monooxygenase.^5^ The anaerobic oxidation of methane (AOM) is mediated by anaerobic methanotrophic (ANME) archaea that can use a variety of electron acceptors: from the energetically less favorable sulfate in marine environments to the more favorable nitrate, humic substances, or metal oxides in brackish and freshwater systems.^6^ Understanding microbial methane oxidation is critical for managing methane emissions and their implications for climate change.^7^

Research on the role of ANME archaea in removing methane is gaining momentum and importance, especially in methane seeps, marine, and freshwater environments including engineered ecosystems such as wastewater treatment plants.^8,9^ ANME-1, ANME-2abc, and ANME-3 are typically found in marine and coastal sediments, where they form consortia with sulfate-reducing bacteria (SRB) to facilitate sulfate-dependent-AOM (S-AOM).^10^ ANME-2d, or “*Ca*. Methanoperedens”, contributes to AOM in anoxic freshwater sediments^11^ from which they have been enriched in laboratory-scale bioreactors.^9,12^ “*Ca*. Methanoperedens” exhibits a versatile metabolism, with different phylotypes described to harbor the potential for nitrate, iron, and manganese reduction.^9,12,13^ In addition, there are growing data correlating sulfate reducers with “*Ca*. Methanoperedens” for S-AOM in meromictic lakes and iron-rich groundwater systems.^14^

The microbial methane filter is exposed to various stressors in the environment, one of which is sulfide exposure through the action of SRB that co-occur in anoxic sediments when freshwater ecosystems are polluted with sulfate from various sources, such as mineral weathering, volcanic activity, wastewater runoff, and sea level changes.^15^ Sulfide inhibits key metabolic processes by damaging copper- and iron-containing cofactors^16^ and inhibiting methanogenesis.^17^ However, detailed studies on the effect of sulfide on freshwater methanotrophs are lacking, and their resilience to sulfide exposure is not well understood, highlighting the need for targeted research. Sulfide oxidation, as well as the oxidation of other reduced sulfur compounds, is phylogenetically widespread and biochemically versatile, resulting in diverse and redundant sulfur oxidation pathways^18^ that are not yet well established for anaerobic methanotrophs. Investigating the reactive sulfur detoxification pathways, inhibitory thresholds, and physiological response of methanotrophs is a crucial step forward and will help to developing accurate models of methane emissions and managing carbon and sulfur cycles in impacted ecosystems.^19^

Here, we employed a “*Ca*. Methanoperedens” bioreactor enrichment culture as an ANME model organism to study the effects of and tolerance to sulfide stress. After short- and long-term exposure to sulfide, we measured the methane oxidation potential via ^13^C–CH_4_ activity assays and followed the bioreactor performance with physicochemical measurements. We investigated the use of specific storage polymers, polyhydroxyalkanoates (PHAs). Additionally, we employed metagenomics and metatranscriptomics to identify which genes and processes respond to sulfide stress to shed light on the possible detoxification mechanisms toward reduced sulfur compounds in anaerobic methanotrophs.

## Materials and Methods

### “*Ca*. Methanoperedens” Enrichment Bioreactor and Medium

The enrichment culture used in this study performs nitrate-dependent anaerobic methane oxidation, with a mixed microbial community dominated by “*Ca*. Methanoperedens BLZ2” sp. and nitrite-scavenging partner “*Ca.* Methylomirabilis oxyfera”.^20,21^ The inoculum used for the original enrichments originated from Twentekanaal (52° 11′04″ N and 6°24′40″ E, The Netherlands), as detailed by Raghoebarsing et al. 2006 ^12^ and further enriched with “*Ca*. Methylomirabilis oxyfera”, as described by Arshad et al. 2015.^20^ The mineral medium used for the microcosm and bioreactor experiments contained (per liter) 240 mg of CaCl_2_·2 H_2_O, 50 mg of KH_2_PO_4_, 160 mg of MgSO_4_·7 H_2_O together with 0.5 mL of trace elements and 0.1 mL vitamins solution composition were employed as specified.^22^ Medium was sparged with Ar/CO_2_ (95:5) during feeding. To avoid iron–sulfur precipitates during the microcosm and bioreactor sulfide pulse toxicity experiments (3 days before the start of “toxicity” and “exposure” activity assays), trace elements excluded iron. Nitrate and nitrite were monitored daily in the bioreactor using MQuantTM test strips (Merck, Darmstadt, Germany). Samples for colorimetric nitrate, nitrite, and ammonium determination measurements were taken several times per week. Ammonium was determined using a high-sensitivity protocol (range from 0.5 to 5 mM) after reaction with 10% orthophthaldialdehyde, as previously described.^23^

### Short-Term Sulfide Batch Experiments

To determine preliminary sulfide toxic thresholds of the anaerobic consortium, we conducted 5 day long microcosm experiments using the culture described above, revealing the short-term response and resilience of the microbial community. We assessed the methane oxidation potential of the biomass at 0 h (control), at 24 h, and at 96 h with two different pulse additions of sulfide, 10 μmol (0.25 mM in final volume of 40 mL) and 20 μmol (0.5 mM in final volume of 40 mL) sulfide exposure. The medium’s pH was buffered with 20 mM HEPES and made anoxic by sparging with N_2_/CO_2_ (95:5) for approximately 2 h. The pH was then adjusted to 7.3 with 1 M KOH. A total volume of 60 mL of biomass from the bioreactor was sampled per biological replicate (*n* = 2 for control incubations and *n* = 3 for experimental conditions) and immediately transferred to the anaerobic chamber in anoxic BD Plastipak 60 mL syringes (Becton Dickinson SA (Madrid, Spain)) that were immediately capped after sample collection to ensure anoxia. To remove residual nitrate and precipitates, the biomass was washed three times with a mineral medium. A total volume of 60 mL was sampled per biological replicate and immediately transferred to the anaerobic chamber in anoxic BD Plastipak 60 mL syringes with capped lids to ensure anoxia. To remove precipitates, the biomass was washed three times with mineral medium. The biomass and fresh mineral medium were then dispensed in a final volume of 40 mL, leaving about 80 mL of headspace. The serum bottles were capped with aluminum crimp caps and red butyl rubber stoppers that were previously boiled twice for 5 to 10 min in 100 mM NaOH and washed twice in water. The bottles were subjected to an additional 5 min N_2_/CO_2_ (95:5) sparging to ensure full anoxic conditions. Serum bottles then received 25 mL of ^13^C–CH_4_ (99%, Cambridge Isotope Laboratories Inc., Cambridge, UK) and 2 mM NaNO_3_, resulting in an overpressure of 1.8 bar in all bottles. Batch incubations were kept in the dark and shaken at 250 rpm at room temperature (Innova 40 orbital shaker, New Brunswick Scientific, USA). Sulfide (Na_2_S × *x*H_2_O) was added at 0.25 and 0.5 mM approximately 2 h after the methane and nitrate addition. The sulfide source used for both batch and bioreactor incubations was sodium sulfide hydrate 60–64% (Na_2_S × *x*H_2_O) (Acros Organics, Thermo Fischer Scientific, The Hague, The Netherlands); the flakes were warmed for 30 min at 60 °C prior to weighing and dissolved in anoxic water. Sulfide concentrations were measured immediately after addition using the methylene blue assay using the HACH 8131 method (1.5–50 μM; HACH, Loveland, CO, USA) using liquid samples retrieved from the bioreactor. ^44^CO_2_ and labeled ^45^CO_2_ were measured in 50 μL headspace samples by gas chromatography–mass spectrometry (GC–MS), using an Agilent 8890 GC System and Agilent 5977B GC/MSD (Agilent Technologies, Santa Clara, CA, USA). A calibration gas mixture consisting of He/O_2_/N_2_/CH_4_/CO_2_/N_2_O with values of (%) balance/1.02/1.03/1.05/1.04/0.050 (Linde Gas Benelux BV, Schiedam, The Netherlands) was used to calculate concentrations. The chromatography data were analyzed using Agilent OpenLab CDS Software. AOM rates were calculated by determining the amount of ^45^CO_2_ over ^44^CO_2_, normalized by the dry mass of the microbial biomass. The positive control was run in parallel to every activity experiment and was set to 100%.

Nitrate and nitrite concentrations were monitored using MQuantTM colorimetric test strips (Merck, Darmstadt, Germany), same as for the bioreactor. When nitrate levels were nearly depleted (2–50 μM), 1 to 3 mM NaNO_3_ was added. Overpressure was constant at 1.6–1.8 bar at all time points and was determined with a Greisinger GMH 3111 Pressure Gauge. The pH was also stable (7.2–7.5) and was monitored before every injection by removal of 250 μL liquid, then measured by a pH meter for microcups (HI-5221, Hanna Instruments, Italy). The butyl rubber stopper was wiped with 70% ethanol before handling.

### Long-Term Sulfide Exposure in Bioreactor

A 2 L bioreactor (Applikon, Delft, The Netherlands) was anoxically inoculated with 2 L of granular biomass from the anaerobic methanotrophs described above. The bioreactor experiment ran for 73 days and was operated as a sequencing fed-batch reactor (SBR). The sequences consisted of a 24 h cycle: 22 h 50 min of medium feed (∼600 mL/day) and sulfide (0.25 mM stock solution added at ∼100 mL/day, during long-term exposure; total addition of sulfide approximately 25 μmol per day), 15 min of settling, 45 min of supernatant removal, and 5 min of buffer time in between cycle changes (total of 10 min), resulting in approximately 3 days of hydraulic retention time. The bioreactor contained two standard six-blade turbines operating at 180 rpm and was maintained at 30 °C with a thermal blanket. Temperature was monitored with a temperature probe and was stable at 30 °C ± 0.5 °C. The pH was buffered with a 100 g/L KHCO_3_ solution and controlled by a BL 931700 pH controller Black Stone (Hanna Instruments, Rhode Island, USA) (Figure S1B). The bioreactor was continuously fed with CH_4_ at a flow rate of 10 mL/min, and loss of volume during the settling/supernatant removal sequence was countered by the addition of Ar/CO_2_ (95/5) through a gas filled buffer bottle to the headspace of the bioreactor to ensure anoxic conditions and avoid negative pressure in the reactor. After an acclimatization period, the bioreactor experiment was divided into three parts: (i) short-term sulfide toxic pulse response (0.5 mM/500 μmol per L bioreactor working volume supplied as shot) (from T0 to T1), (ii) sulfide long-term exposure period (0.25 mM/day) for approximately 6.5 weeks, and (iii) second toxic pulse response (0.5 mM) after acclimatization (from T2 to T3) (Figure 1). The methane oxidation rate was determined during the three periods: control (T0), toxicity (T1), and exposure (T3). The ^13^C–CH_4_ activity assays were performed with the entire bioreactor and lasted 5 days. During the test, the bioreactor was operated in the batch mode with 20% ^13^C–CH_4_ and additional N_2_ in the headspace to achieve an overpressure of 1.2–1.4 bar. The 0.5 L headspace was first flushed for 2–3 h with Ar/CO_2_ (95:5) to remove residual methane traces, and stirring was increased to 250 rpm to allow for increased methane diffusion. The initial nitrate concentration was approximately 1–1.5 mM. Over the 5 days, labeled ^13^C–CO_2_ and ^12^C–CO_2_ were measured by GC–MS as aforementioned. Sulfide was measured immediately at specified time points before and after DNA sampling (T0–T1 and T2–T3) (Figure 1). The dry weight (*n* = 3, 10 mL) of the biomass used was determined after drying to constant weight for 3 days at 100 °C.

**Figure 1:**
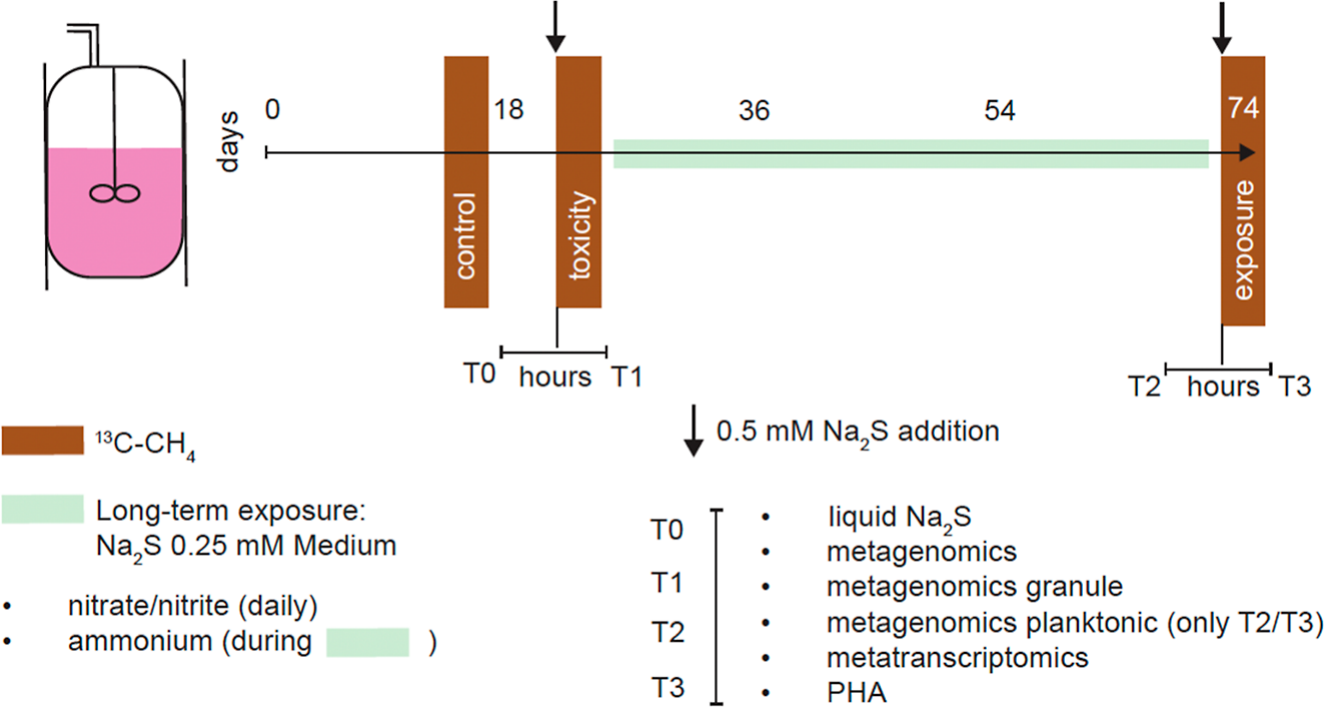
Bioreactor sulfide toxicity experiment workflow. Whole bioreactor activity assays with labeled ^13^C–CH_4_ are indicated in brown (4–5 days). A duration of 4–5 h passed between T0–T1 and T2–T3. Sulfide additions are indicated by an arrow (spike, 0.5 mM Na_2_S) or green rectangle (acclimation period of 6.5 weeks, 0.25 mM/day).

### Bioreactor DNA and RNA Sampling

To track the microbial community and activity over time, DNA and RNA samples were collected at four different time points for downstream metagenomics and metatranscriptomics, respectively (Figure 1). The first two time points, T0-T1, were collected immediately before and 2 h after the first 0.5 mM sulfide spike. The latter two points, T2–T3, were collected after the longer sulfide exposure period (∼6.5 weeks), immediately before and after 2 h of 0.5 mM (500 μmol per L bioreactor) sulfide addition (Figure 1). To characterize the granular vs free-living morphotype of “*Ca.* Methanoperedens”, biomass was vacuum filtered at time points T0, T1, T2, and T3 using hydrophilic polycarbonate membranes (5.0 μm and 47 mm diameter) (TMTP04700) (Millipore, Darmstadt, Germany). DNA was extracted once, whereas triplicate extractions were performed per time point for RNA, to allow for the construction of metagenome-assembled genomes from DNA and the statistical analysis of transcriptomes. Biomass was immediately stabilized upon sampling by mixing 2 mL of biomass with 6 mL of PowerProtect DNA/RNA solution (1:3) (Qiagen Benelux B.V., Venlo, The Netherlands). The stabilized mixture was spun down, and the remaining pellet was freeze-dried overnight and stored at −70 °C. DNA extractions were performed using the DNeasy PowerSoil Kit (Qiagen, Hilden, Germany), and RNA was extracted with the RNeasy PowerSoil Kit (Qiagen, Hilden, Germany), with initial manual pottering of samples to disrupt the granules. DNA and RNA quality was determined using a NanoDrop Spectrophotometer ND-1000 (Isogen Life Science, Utrecht, Netherlands) and a Bioanalyzer 2100 (Agilent, Santa Clara, CA, USA), respectively. Concentrations were measured with a Qubit 2.0 fluorometer using the DNA dsDNA HS kit for DNA and the RNA HS kit for RNA (Thermo Fisher Scientific, Waltham, MA, United States). For the T2 and T3 planktonic (filtrate fraction) DNA samples, the AMPpure XP (Beckman Coulter, CA, USA) DNA purification kit was employed to obtain a higher DNA quality and yield of these low biomass samples. The planktonic fraction of time points T0 and T1 yielded insufficient DNA for downstream library preparations. All RNA samples included an RNAase-Free DNAase treatment (Qiagen, Hilden, Germany). Sequencing was performed by Macrogen Europe BV (Amsterdam, The Netherlands).

### Metagenomics

Metagenomic sequencing was performed with a TruSeq DNA PCR free library using an insert size of 550bp on a NovaSeq6000 (Illumina) platform, producing 2 × 151bp paired-end reads (10 Gbp/sample). Read quality was assessed with FASTQC version 0.11.9 before and after quality trimming, adapter removal, and contaminant filtering, performed with BBDuk (BBTools v38.75). Trimmed reads were coassembled *de novo* using metaSPAdes v3.14.1 ^24^ and mapped to assembled contigs using BBMap (BBTools v38.75).^25^ Contigs at least 1000-bp long were used as template for read mapping of metatranscriptomic sequences as well as for binning. Sequence mapping files were handled and converted using Samtools v1.10., later used for binning with CONCOCT v2.1,^26^ MaxBin2 v2.2.7,^27^ and MetaBAT2 v2.12.1.^28^ Resulting metagenome-assembled genomes (MAGs) were dereplicated with DAS Tool v1.1.1 ^29^ and taxonomically classified with the Genome Taxonomy Database Toolkit GTDB-Tk v2.1.0.^30^ Metagenomic mapping statistics were generated via CheckM v1.1.2.^31^ For a metagenomic binning taxonomical read-recruitment assessment, SingleM v0.16.0 (https://github.com/wwood/singlem) was employed. MAG completeness and contamination were estimated with CheckM2 v1.0.1.^32^ Metagenome-assembled genomes were annotated with DRAM v1.0,^33^ and with default options, except min_contig_size at 1000 bp, and METABOLIC v4.^34^ Additional genes of interest were searched via BLASTp and HMM analyses. To corroborate poorly annotated genes/proteins, we opted to validate manual curations with the NCBI Batch Entrez Conserved Domains search option and InterPro^35^ web browers’ search option.

To obtain a read-based “*Ca.* Methanoperedens” granular and free-living relative abundance, an additional shallow metagenome was generated. Library preparation of the metagenome was performed using the Nextera XT kit (Illumina, San Diego, CA, USA) according to the manufacturer’s instructions. Enzymatic tagmentation was performed starting with 1 ng of DNA, followed by incorporation of the indexed adapters and amplification of the library. After purification of the amplified library using AMPure XP beads (Beckman Coulter, Indianapolis, USA), libraries were checked for quality and size distribution using the Agilent 2100 Bioanalyzer and the high sensitivity DNA kit. Quantitation of the library was performed by Qubit using the Qubit dsDNA HS Assay Kit (Thermo Fisher Scientific Inc. Waltham USA). The libraries were pooled, denatured, and sequenced with a MiSeq (Illumina) sequencer (San Diego, CA, USA). Paired end sequencing of 2 × 300 bp was performed using the MiSeq Reagent Kit v3 (San Diego, CA, USA) according to the manufacturer’s protocol yielding 1.6–1.7 Gbp for the granular fraction and 0.3 or 1.1 Gbp for the T3 and T3 planktonic fractions, respectively.

### Metatranscriptomics

Metatranscriptomic sequencing was performed using a TruSeq stranded with NEB rRNA depletion kit (bacteria) (Illumina, San Diego, CA, USA) on a NovaSeqX 10B (Illumina) platform, generating 150-bp paired-end reads with ∼15 Gb throughput/sample. Raw sequences were quality trimmed using sickle v1.33 (https://github.com/najoshi/sickle) and rRNA contaminant-filtered, mapped against the DRAM-generated scaffolds, and transcripts per million (TPM) values were generated using trancriptm v0.4 (https://github.com/sternp/transcriptm). Differential expression (log2foldchain and p-adjusted) was evaluated using the DESeq2 library in R.^36^

### Polyhydroxyalkanoates (PHAs) Quantification

The PHA amount in the “*Ca.* Methanoperedens” sp. enriched biomass was measured at the same time points as the genomic samples were taken (Figure 1). The PHAs were hydrolyzed to polyhydroxy acids, and these acids were then methylated. These methylated (poly)­hydroxy acids were analyzed by GC–MS. Refer to Echeveste Medrano et al. 2024 ^37^ for detailed extraction protocol.

## Results and Discussion

### Methane Oxidation by “*Ca.* Methanoperedens” is Inhibited by Sulfide Exposure

Batch incubations (“Batch”) were used to assess the short-term sulfide inhibitory effect and resilience on the “*Ca.* Methanoperedens BLZ2” sp. enrichment using two sulfide pulses of 0.25 and 0.5 mM as stressors (Figure 2). At 0.25 mM sulfide exposure, we observed a drop of approximately 20% in activity within 24 h but a recovery to 125% within 5 days. At 0.5 mM sulfide, the activity was reduced to just 20% of the control, and after 5 days, the activity was 70% compared to the control (Figure 2). Follow-up bioreactor experiments used 0.5 mM sulfide as the toxic threshold, whereas 0.25 mM was used for long-term exposure.

**Figure 2:**
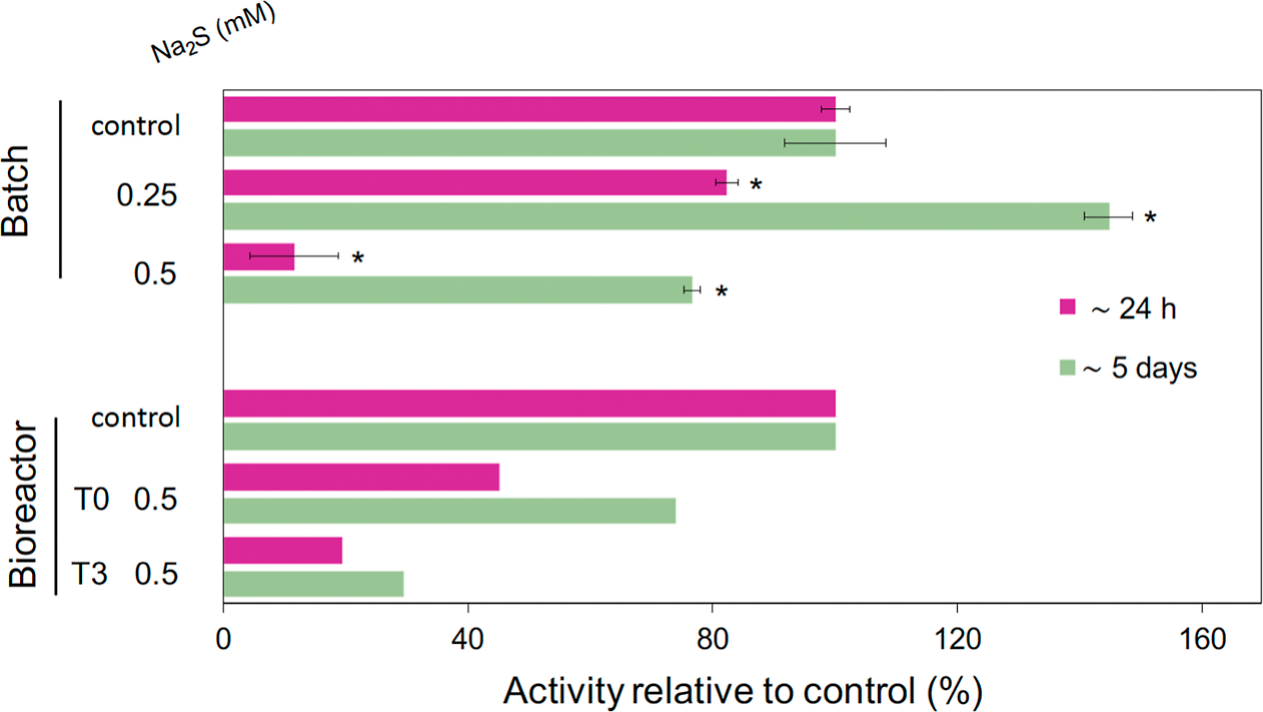
Methane oxidation potential in the microcosm experiment (batch, *n* = 2 for control and *n* = 3 for experimental treatments) or bioreactor experiments (*n* = 1) is expressed as activity relative to the control (either batch or bioreactor). Relative activity was calculated either by comparing the first 24 h, or during the entire monitoring period of 5 days. Methane oxidation potential was calculated by cumulative ^45^CO_2_/^44^CO_2_ normalized by dry weight (g). Significant differences (two tailed, heteroscedastic *t*-test) of the different incubations compared to the control are indicated with an asterisk (*P* < 0.05). For the microcosm experiment, the bar indicates standard deviation. The average cumulative ^45^CO_2_/^44^CO_2_ g^–1^ day^–1^ for the bioreactor and batch controls were 2.04^–2^ and 1.21^–5^ ± 1.00^–6^, respectively.

Briefly, in the long-term sulfide exposure experiment, the bioreactor (Figure 1) was spiked with 0.5 mM sulfide, followed by a daily 0.25 mM spike for 6.5 weeks and followed by a second 0.5 mM spike. Exposure of the bioreactor biomass to 0.5 mM sulfide resulted in a drop to about 50% of the original activity at 24 h with a recovery to about 70% at 5 days. Surprisingly, the long-term exposure at approximately 0.25 mM/day did not help the culture adapt to sulfide exposure. With the second 0.5 mM sulfide pulse (Figure 2), the activity dropped to only 25% with a slight recovery to 33% after 5 days compared with the control.

Nitrate was almost always fully consumed during the monitoring period, with some short-term accumulation (200–800 μM) during long-term sulfide exposure (Figure S1A). Nitrite was mostly below the detection limit, except on days 18, 28, and 66 when 200–800 μM nitrite was detected (Figure S1A). In line with previous studies, “*Ca.* Methanoperedens” experiments showed that dissimilatory nitrate reduction to ammonium occurred, and ammonium concentrations ranged from 200 to 1200 μM (Figure S1A). Sulfide concentrations dropped below the detection limit within 2 h after their addition during both spikes and the long-term exposure period.

Our experiments showed a strong inhibition of ∼45% (bioreactor) and ∼12% (batch) of methane oxidation activity of the anaerobic consortium with the methanotroph “*Ca.* Methanoperedens” after a 0.5 mM sulfide spike, which recovered to ∼74% and ∼77% after 5 days, respectively (Figure 2). These inhibitory thresholds contrast with those reported for brackish ANME S-AOM, where ∼1 mM sulfide inhibited 50% of AOM activity.^38^ However, for the measured S-AOM sulfide-inhibition, the *in situ* sulfide was not converted and remained stable during the experiment, as opposed to our incubations where the sulfide was quickly disappearing (Figure S1B). Considering the sulfide inhibition after repeated long-term sulfide exposure in the “*Ca.* Methanoperedens” enriched culture (0.25 mM/day), the tolerance reduces to ∼20% (24 h rate) or ∼30% (5 day recovery rate) after the second 0.5 mM sulfide addition. As sulfide rapidly reacts with Fe­(II), part of the sulfide might be inorganically removed through reaction with residual Fe­(II). Concomitant iron starvation might contribute to the toxicity of sulfide to “*Ca.* Methanoperedens” in the bioreactor and in the environment as they rely on numerous iron-containing *c*-type cytochromes for their metabolism.^39^ Overall, our results clearly show that sulfide has a much stronger inhibitory effect on nitrate-dependent methane oxidation, as opposed to sulfide-dependent methane oxidation, even though sulfide is rapidly removed after addition to the nitrate-dependent methane oxidizing microbial community.

Overall, this suggests that the freshwater “*Ca.* Methanoperedens” is less tolerant to sulfide exposure compared to brackish/marine ANME. Such differential sulfide tolerance has also been observed within marine ANME species, with ANME-1 remained active under higher sulfide concentrations while ANME-2ab activity was negatively correlated with sulfide.^40^

The presence and transcriptional activity of “*Ca.* Methanoperedens” are not negatively impacted by sulfide exposure.

To follow changes in the microbial community composition and expression patterns, we sequenced both DNA and RNA at T0, T1, T2, and T3 (Figure 3). “*Ca.* Methanoperedens” dominated the overall community in both DNA and RNA fractions throughout the reactor run (T0, T2, and T3) (Figure 3). The rare biosphere (“Others” in Figure 3) was hardly represented in the transcriptome, although they made up a significant portion (between 25% and 40%) of the DNA reads, indicating slow mineralization of decaying biomass, usage of exudates, or just dormant yet not dead.

**Figure 3:**
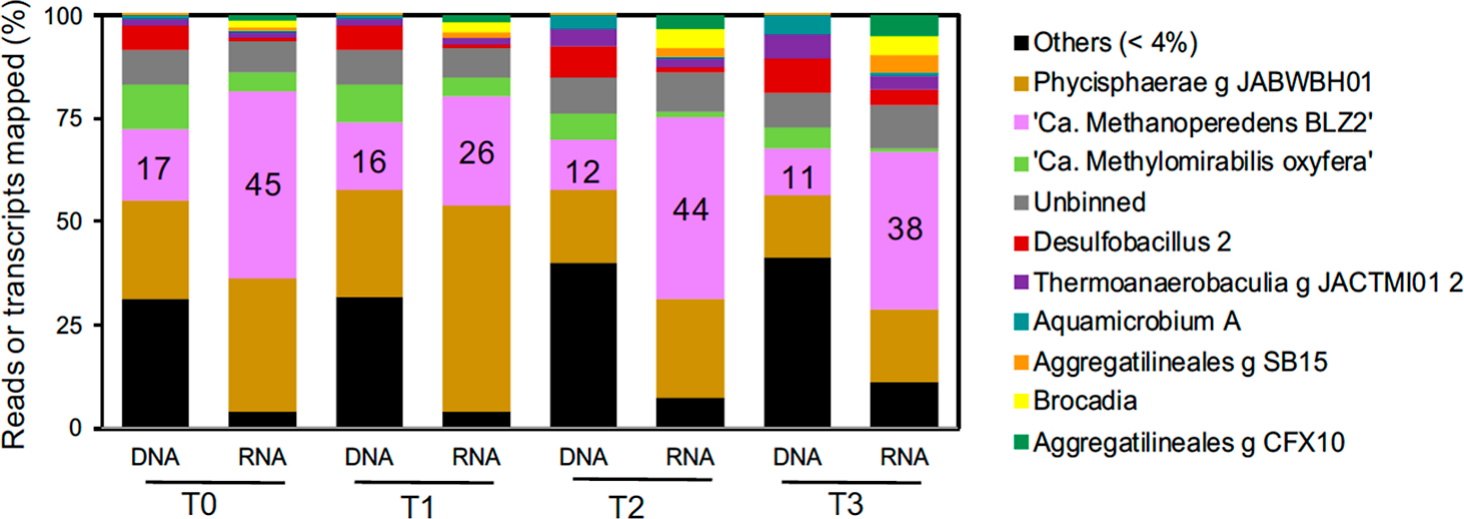
Top MAGs (>4% metagenomics reads assigned for all time points) community and activity indicated as TPM (>4%). Additional categories include “Others (<4%)” for MAGs that were below the 4% thresholds and unbinned fraction. Categories are ordered from highest to lowest on DNA-based % of T0 values. “*Ca.* Methanoperedens” percentages are indicated with numbers in the pink box in addition.

Regarding the general microbial community composition change, we observed that MAGs “Desulfobacillus 2”, “Thermoanaerobaculia g JACTMI01 2”, “Aquamicrobium A”, “Aggregatilineales g SB15 & g CFX10”, and “Brocadia” anammox bacteria, among others, showed a general upward trend in both metagenomics and metatranscriptomics (Figure 3, Tables S1 and S2). In contrast, the methane-oxidizing partner and nitrite-scavenger “*Ca.* Methylomirabilis oxyfera” reduced its overall transcription after long-term exposure and sulfide spikes (Figure 3).

Despite the shifts in methane oxidation potential, “*Ca.* Methanoperedens” demonstrated high resilience, as previously reported for oxygen or salt stress.^37,41^ Notably, “*Ca.* Methanoperedens” remained the most active community member (RNA-based) for all conditions except for T1, where “Phycisphaerae g JACWBH01” represented almost 50% of the transcripts recovered (Figure 3). We previously analyzed MAG “Phycisphaerae g JACWBH01” and reported its most likely function as the heterotrophic nitrate reducer, which is congruent with the genomic content of the MAG in this study. The bloom of *Phycisphaerae* activity under sulfide stress at T1 suggests that available cell debris or excreted metabolites that are formed because of acute sulfide stress favor the lifestyle of Phycisphaerae enabling them to potentially outcompete “*Ca.* Methanoperedens” for nitrate under stress.

The main nitrite-scavenging microbial community member, “*Ca.* Methylomirabilis oxyfera”, was previously reported to contribute about 30% to the overall methane oxidation rate in this culture (in batch activity assays) and was contributing to an additional 40% of AOM activity through syntrophy with “*Ca.* Methanoperedens BLZ2” in a culture that at the time showed a relative abundance of 28% “*Ca.* M. oxyfera” and 19% “*Ca.* Methanoperedens BLZ2”.^42^ In our study, “*Ca.* M. oxyfera” was less abundant at the start of the experiment with 11% relative abundance compared to a similar abundance of “*Ca.* Methanoperedens BLZ2” of 17%. “*Ca.* M. oxyfera” showed a decrease in relative abundance and activity in the DNA and the RNA data throughout the experiment, as opposed to “*Ca.* Methanoperedens BLZ2”, indicating inhibition of this methanotroph due to sulfide exposure and toxicity. Consequently, the inhibition of methane oxidation activity observed for this culture, especially upon long-term sulfide exposure, is probably due to activity inhibition of both anaerobic methanotrophs, a gradual loss of “*Ca.* M. oxyfera” from the enrichment culture, and the associated impact on the syntrophic relationship (Figure 3).^42,43^ Therefore, while methane oxidation is profoundly affected by sulfide inhibition, the relative rates of inhibition for “*Ca.* Methanoperedens BLZ2” cannot be derived from the data presented. Investigating “*Ca.* Methanoperedens” cultures with a different nitrite scavenger (e.g., anammox bacteria or *Phycisphaerae*) might elucidate this knowledge gap.

Genes encoding core metabolic enzymes of “*Ca.* Methanoperedens” are upregulated at the first sulfide spike but get downregulated after long-term exposure.

The general response of “*Ca.* Methanoperedens” was assessed by its genes and comparing them between the three time points: T0 vs T1, T0 vs T2, and T0 vs T3. A total of 202, 30, and 321 genes were significantly upregulated, and 265, 67, and 479 genes were downregulated (Padj <0.05) with more than 2 log_2_FC between T0 vs T1, T0 vs T2, and T0 vs T3, respectively (Table S2).

We then assessed the response of the transcripts belonging to the methane-oxidizing complex methyl coenzyme-M reductase (MCR) and nitrate reductase to the different sulfide exposures (Tables 1 and Table S3). The *mcr* genes were upregulated (*mcrA*, log_2_ FC 2.31) only during the first sulfide spike but were downregulated during long-term exposure (*mcrA*, log_2_ FC −0.59) and after the second pulse (*mcrA*, log_2_ FC −2.31) (Table 1). The “*Ca.* Methanoperedens” nitrate reductase (*narG*) gene showed opposite transcriptional trends to the *mcr* genes. Genes encoding for Mcr of “*Ca.* Methanoperedens” were upregulated from T0 to T1 congruent with less severe inhibition of methane oxidation activity after the first pulse of sulfide addition. In contrast, the loss of activity over long-term sulfide exposure is reflected in the downregulation of key metabolic genes at that time point (Figure 2 and Table 1). Although the genes of the MCR complex show reduced expression, this does not indicate a decreased survivability. Instead, it likely reflects a metabolic shift for adaptation, similar to what was observed in a salt-stress experiment with “*Ca.* Methanoperedens”^37^ where the metabolism shifted toward osmoregulation to help the culture adapt to marine salinities.

**Table 1:**
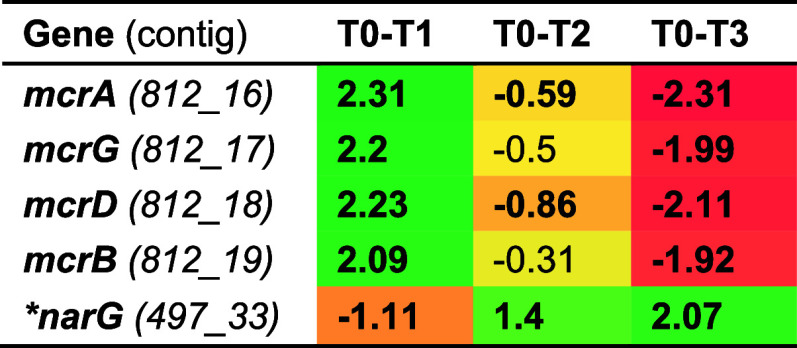
Methyl-coenzyme M Reductase (MCR) Complex and Nitrate Reductase (*narG*) Gene Transcript Expression from “*Ca.* Methanoperedens”^a^

a Methyl-coenzyme M reductase alpha subunit [EC:2.8.4.1] (*mrcA*), methyl-coenzyme M reductase gamma subunit [EC:2.8.4.1] (*mcrG*), methyl-coenzyme M reductase subunit D (*mcrD*), methyl-coenzyme M reductase beta subunit (*mcrB*) [EC:2.8.4.1]. Padj <0.05 is indicated in bold. Asterisks indicate genes belonging to the unbinned fraction that were later taxonomically classified. Condition differences (T0–T1, T0–T2, and T0–T3) indicate log 2 fold chain (FC) values. “NA” (not available).

### Rare Phyla Apparently Detoxified Sulfide via Sqr and FccAB during the First Sulfide Exposure (T0–T1)

To determine the potential for sulfide detoxification and oxidation across conditions, we investigated the sulfide quinone oxidoreductase transcripts (sqr) across all MAGs (Table S3).

We observed that the rare microbial community, that is, not belonging to the top >4% MAGs (Figure 1), was most likely responsible for the most significantly changed (Padj <0.05 and log 2 FC min 2) transcripts for the first step of sulfide oxidation (Table 2). MAGs “Nordella 1”, “Hyphomicrobium 1”, “Burkholderiales g SHXO01”, “Rubrivivax”, “Xanthobacteraceae”, and others probably catalyzed the first step of sulfide oxidation from T0 to T1. Conversely, from T0 to T2 (downward trend, nonsignificant) and T0 to T3 (mostly significant), almost all MAGs, except for “Hyphomicrobiaceae g AWTP1 13”, showed lowered expression values for *sqr*.

**Table 2:**
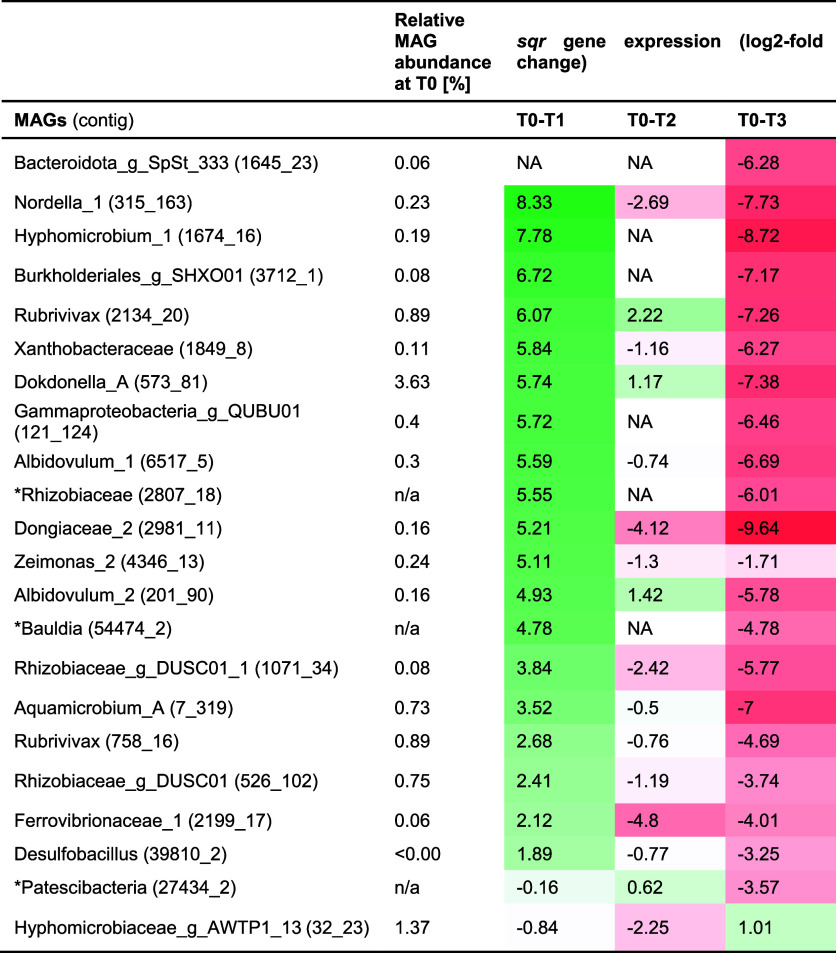
Sulfide/Quinone Oxidoreductase (sqr) [EC: 1.8.5.4] Gene Expression across all Conditions in MAGs That Showed a Significant Change of Padj <0.05 (in Bold) and log 2 FC of 2 Up- or Downregulated for at Least One Condition^a^

a MAGs have been ordered from the highest to lowest level of expression based on T0–T1 condition. Asterisks indicate genes belonging to the unbinned fraction that were later taxonomically classified. Condition differences (T0–T1, T0–T2, and T0–T3) indicate log 2 FC values. “NA” (not available).

We also considered sulfide detoxification/oxidation by the contiguous cytochrome subunit of sulfide dehydrogenase (*fccA)* and sulfide dehydrogenase [flavocytochrome c] flavoprotein chain (*fccB)* [EC:1.8.2.3] gene presence and transcript expression across all conditions and MAGs (Table S3) (Padj <0.05 and log2FC min 2). Here, we observed trends similar to those of *sqr*, with general upregulation trends from T0–T1 vs downregulation from T0–T2 and T0-T3. MAGs “Desulfobacillus 2” and “Casimicrobiaceae g JACPUX01” *fccA* showed increased significant expression from T0–T1 and T0–T3, respectively (Table S4). The rest of the *fccB* or *fccA* transcripts were downregulated in almost all MAGs with significant expression from T0–T2 and T0-T3.

All in all, the sulfide scavenging was most likely carried out by the rare community members indicated by upregulation of *sqr* and *fccAB* during the first sulfide exposure (Tables 3 and Table S4) with a general downregulation after long-term exposure for the same 0.5 mM sulfide pulse (Figure 2). This observation is suggestive of the overall response of the community toward more efficient processes or alternative sulfur metabolism pathways. One example is the sulfite reductase that might be cycling sulfur through assimilatory or dissimilatory pathways.^44,45^

**Table 3:**
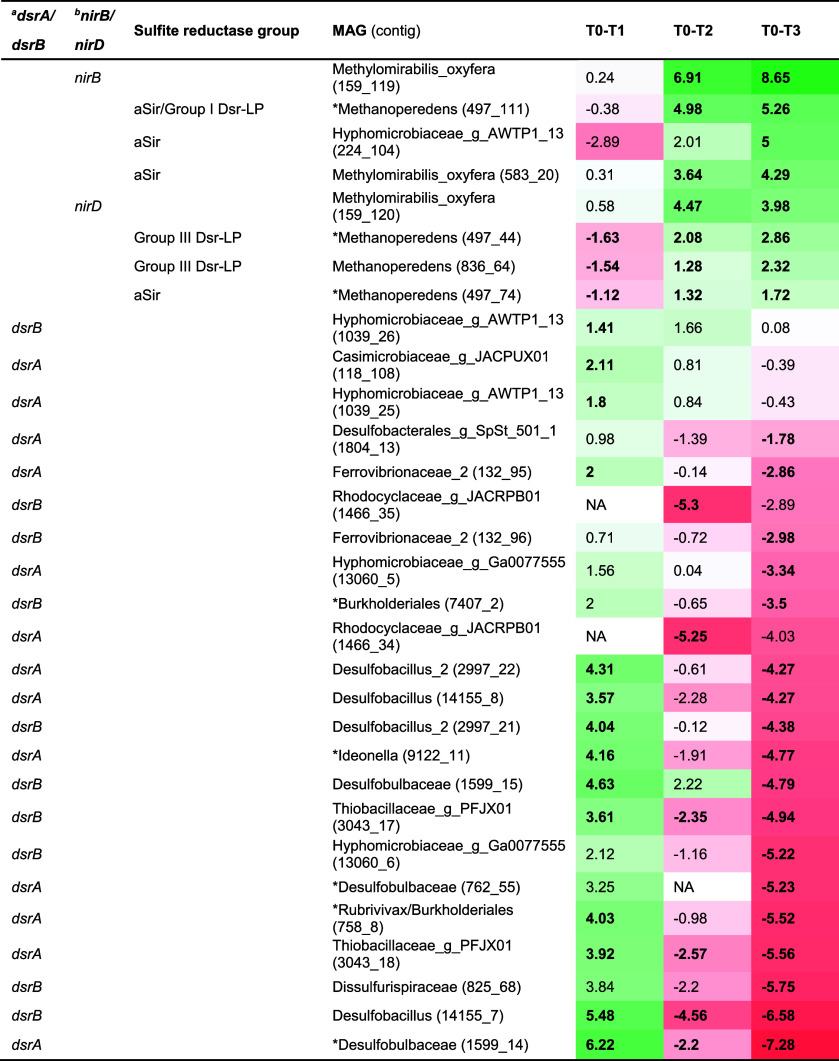
“Nitrite and Sulfite Reductase 4Fe–4S Domain Containing Protein” (PFAM: PF01077.27) Transcript Expression^a^

a Transcripts that showed Padj <0.05 for at least one condition were included for the analysis and indicated in bold. Genes were classified based on their KEGG number or INTERPRO id (for sulfite reductases): ^a^ *dsrA* (K11180) & *dsrB* (K11181); ^b^ *nirB* (K00362) & *nirD* (K00363); ^c^assimilatory sulfite reductase (aSir) (IPR051329), aSir/Group I Dsr-LP (IPR045854), or Group III Dsr-LP (IPR045169, IPR045854). We indicated an asterisk for the gene found in the *unbinned fraction and/or when manually assigned to a MAG. Sulfite reductases were classified as in Yu et al. (2018). Transcripts were ordered from highest to lowest expression change from T0–T3. Condition differences (T0–T1, T0–T2 and T0–T3) indicate log 2 FC values. “NA” (not available).

### Methanotrophic Community Activated Sulfite Reductase after Long-Term Sulfide Exposure (T2 and T3)

We screened for sulfite detoxification potential by a query search of “Nitrite and sulfite reductase 4Fe–4S domain” (PFAM: PF01077.27) protein family genes in our metagenome (Table S3). We found a total of 33 sulfite/nitrite reductase genes that were significantly up- or downregulated upon sulfide exposure (Table 3). These sulfite/nitrite reductases were divided into four categories: dissimilatory sulfite reductase alpha/beta subunit (DsrAB), nitrite reductase large/small subunit (NirBD), assimilatory sulfite reductase (aSir) (IPR051329), aSir/group I dissimilatory reductase-like protein (aSir/Dsr-LP) (IPR045854), and group III Dsr-LP (IPR045169) of unknown physiological function.

We found a marked increase and subsequent decrease for *dsrAB* expression during the first sulfide pulse and subsequent time points, coinciding with increased *sqr* and *fccAB* expression, respectively (Tables 3 and S4), in the nonmethanotrophic microbial community. In “*Ca.* Methanoperedens BLZ2” and “*Ca.* M. oxyfera”, group I Dsr-LP, aSir, and group III Dsr-LP increased in expression between T0–T2 and T0–T3 (Table 3).

We also checked for additional mechanisms related to sulfur cycling present in “*Ca.* Methanoperedens BLZ2” and “*Ca.* M. oxyfera”. This search detected genes annotated as sulfate adenylyltransferase (*sat*) (KEGG: K00958) to both MAGs and l-cysteine S-thiosulfotransferase (*soxX*) (KEGG: K17223) only to “*Ca.* M. oxyfera” that were upregulated at T2 and T3 (Table S5).

Our study highlights the relevance of sulfite reductases upon long-term exposure to sulfide (Table S4). These enzymes are known to protect methanogens from sulfite inhibition.^44,46^ While sulfide gets incorporated into the biomass, sulfite builds up inside the cell reacting with nucleic acids, proteins, and enzyme cofactors.^47^ We identified two significantly upregulated sulfite reductases belonging to group III Dsr-LP exclusive to “*Ca.* Methanoperedens” and not present in marine ANME.^38^ Recently, combined transposon library construction with high-throughput growth studies on model methanogen *Methanococcus maripaludis* showed that the group III-Dsr-LP allowed for sulfite resistance in the presence of sulfite as a sulfur source.^48^ On the contrary, marine ANME were hypothesized to employ group II coenzyme F_420_-dependent sulfite reductase (Fsr), a protein found to be more abundantly present in the metaproteome of marine ANME^45^ and hypothesized to confer sulfite detoxification potential in brackish ANME-2.^38^ Group II Fsr has not been found in “*Ca.* Methanoperedens” MAGs.^45^ Similar to Group III-Dsr-LP, group I Fsr was first described to be highly expressed under sulfite as a sole sulfur source and provided detoxification potential in *Methanocaldococcus jannaschii*.^49^ The distinct presence of different groups of sulfite reductases in freshwater and marine ANME could explain the differential acclimation to sulfide exposure.

Another area for future studies is the possible dual role of “*Ca.* Methanoperedens” group III Dsr-LP not only as a sulfite reductase but also as a nitrite detoxification mechanism. During our long-term exposure to sulfide, nitrite accumulated in the bioreactor (Figure S1A). The simplest sulfite reductase structure, belonging to the group I Fsr sulfite reductase, was extracted and crystallized from *Methanothermococcus thermolithotrophicus* together with follow up enzymatic assays, indicating a higher nitrite (*K*_m_ = ∼2.5 μM) over sulfite (*K*_m_ = ∼15.5 μM) preference.^50^ Furthermore, Jespersen et al. (2023) analyzed the binding pocket for sulfite and observed that group II Fsr has a larger binding pocket than that of the analyzed group I Fsr. This observation led the hypothesis that group II Fsr might harbor a different substrate specificity. Similarly, to characterize the catalytic activity of group II Fsr in ANME, a purified recombinant ANME-2c enzyme expressed in *Methanosarcina acetivorans* was employed, leading to the discovery that group II Fsr showed nitrite reductase activity but no activity toward sulfite or thiosulfate.^51^ This suggests that group II Fsr could be conferring nitrite detoxification potential to marine ANME. “*Ca.* Methanoperedens” possesses the nitrite reductase NrfAH to overcome nitrite toxicity which could explain the absence of group II Fsr encoded in the genome.^20,52^

The described upregulated group III Dsr-LP sulfite reductase gene is phylogenetically distinct from other Fsr groups and most closely related to AsrC, which is distinct both from dissimilatory DsrAB and also aSir.^45^ AsrC has been described to work as a dissimilatory sulfite reductase,^53^ yet it has also been reported that *asrABC* was upregulated under growth with nitrate, and not sulfate, in the acidophilic sulfate reducer *Acididesulfobacillus acetoxydans*.^54^ No ANME or methanogens have been reported to harbor *asrC*,^45^ however, another study recently described group III Dsr-LP sulfite reductases in high-quality “*Ca.* Methanoperedens” MAGs resolved from freshwater meromictic lake Cadagno sediment.^55^ In those ecosystems, the role of group III Dsr-LP sulfite reductases for sulfite detoxification seems more probable over nitrite removal, given the low availability of nitrate/nitrite and particularly the high availability of sulfate.^55^ To disentangle the nitrite/sulfite preferences of “*Ca.* Methanoperedens” group III Dsr-LP, further protein purification and enzymatic assays to determine the preferences for either sulfite or nitrite are needed.

### PHA and (De)­granulation upon Long-Term Sulfide Exposure

To investigate whether “*Ca.* Methanoperedens” would shift aggregation levels or use its storage compounds to counteract sulfide stress, we determined the amount of PHA per biomass and the number of planktonic cells. We observed a reduction in the PHAs especially after long-term exposure to sulfide (Figure 4). The expression of genes encoding proteins responsible for the degradation of PHA did not change significantly (Table S6). Previous research on bacteria and haloarchaea has demonstrated that PHA metabolism is regulated mostly post-translationally by competitive inhibition or phosphorylation of key enzymes,^56^ which explains the lack of transcriptional response even though PHA metabolism was clearly impacted in our study (Figure 4). There is no research on close relatives of “*Ca.* Methanoperedens”; hence, the exact mechanism of PHA regulation remains unclear.

**Figure 4:**
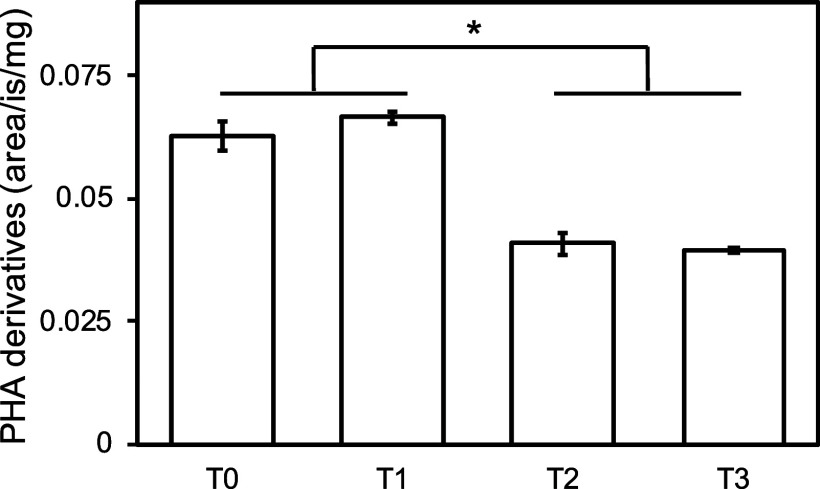
Polyhydroxyalkanoate (PHA) derivatives measured by gas chromatography–mass spectrometry (GC–MS) across the four different conditions (*x*-axis). PHA derivative quantities were normalized using an internal standard and dry weight (mg). Significant differences (two tailed, heteroscedastic *t*-test) are indicated with an asterisk (*P* < 0.05).

PHAs have been previously described as “*Ca.* Methanoperedens” storage polymers.^37,45,57,58^ Concomitant with previous salt stress experiments on “*Ca.* Methanoperedens”, we observed PHA reduction upon 6.5 weeks of sulfide exposure (Figure 4).^37,58^ Nitrate-dependent PHA usage could therefore function as a stress defense mechanism saving the methane oxidation (reduced MCR gene expression from T0–T2 and T0–T3) for energy metabolism (Table 1 and Figure 4).

### DNA and RNA-Based Indications of Morphotype Shift upon Long-Term Sulfide

Our investigation included the monitoring of “*Ca.* Methanoperedens” morphotypes during the various exposure periods. Previous salt-stress experiments transcriptional shifts and DNA-biomass fractionation of granulated versus suspended cells were described.^59^ In our study, we first filtered the “*Ca.* Methanoperedens” biomass into two fractions: retentate (granular) and filtrate (planktonic) (Figure 1). We were unable to obtain enough DNA for the planktonic fraction at time points T0 and T1 for metagenomic sequencing, whereas samples at T2 and T3 gave high enough values to proceed (Figure S3). Read-based classification of the granular fraction of “*Ca.* Methanoperedens” indicated a drop from 20% to 11% of “*Ca.* Methanoperedens” (from T0 to T2), with no major changes from T2 to T3 (16–18%). We observed a minimal fraction of “*Ca.* Methanoperedens” in the planktonic fraction of T2 and T3, constituting about 0.5–0.9% of the total reads recovered (Figure S3).

We detected different RNA-seq trends on the granular “*Ca.* Methanoperedens”-specific cell division transcripts (*ftsZ*) and archaeal flagellin (*flaF*/*flaH*/*flaI*/*flaJ*) between control (T0) and T1 vs control (T0) and T2 or T3 (Tables 3 and 7). Moreover, the general morphotype shift gene marker expression increased upon the first sulfide spike (Table 7). This distinct morphotype gene marker expression shift in the different time points analyzed (Table 7) showed a degree of similarity with the increase in markers for morphotype shift in “*Ca.* Methanoperedens” under oxygen and salt stress.^37,41^

## Implications and Future Work

This work presents the first physiological study on freshwater ANME “*Ca.* Methanoperedens” exposed to sulfide stress. We highlight a marked methane oxidation activity drop at 0.5 mM sulfide together with an increase in expression of “*Ca.* Methanoperedens” sulfite reductases (group III Dsr-LP) as a putative sulfite detoxification mechanism upon long-term exposure. In addition, the potential usage of PHAs together with sulfide scavenging community members appears to help enable “*Ca.* Methanoperedens” survival under sulfide stress. Future work on nitrite, salt, and sulfide stressors on anaerobic methanotrophs could benefit from the analysis of the response of distinct sulfite reductases to such stressors. Our study contributes to an improved understanding of stress response in ANME archaea, particularly relevant in the context of eutrophic natural or engineered ecosystems.

## Supplementary Material





## Data Availability

Presented 16S rRNA gene amplicon and metagenomics data are available under European Nucleotide Archive project number PRJEB81701.

## Notes

Physicochemical data of the bioreactor experiment; single M read-based metagenome analysis quality based on recovery of taxonomical marker S3; microbial community analysis of granular and planktonic fractions; completeness, contamination, and bin size of all retrieved GTDB-tk classified metagenomic-assembled genomes (MAGs) and their abundance; report on treated metatranscriptomic data (all TPM values recovered per MAG and contig); DRAM-annotated MAGs originating from coassembly of all metagenomic sample points; transcriptional response of *fccA* and *fccB* in various members of the microbial community; additional sulfur cycling gene transcript expression across all conditions in MAGs affiliated with methane oxidizers; and transcriptional response of “*Ca.* Methanoperedens” PHA cycling marker genes and morphotype shift gene markers (PDF)
Completeness, contamination, and bin size of all retrieved GTDB-tk classified MAGs and their abundance; report on treated metatranscriptomic data (all TPM values recovered per MAG and contig); and DRAM annotated MAGs originating from co-assembly of all metagenomic sample points (XLSX)
