# Supplementary material for: Physiological Stress Response to Sulfide Exposure of Freshwater Anaerobic Methanotrophic Archaea
Source: Environ Sci Technol. 2025 May 19;59(21):10262–73. doi: 10.1021/acs.est.4c12489 (PMC12138975; doi:10.1021/acs.est.4c12489)
Supplement: Supplementary file 1 [file es4c12489_si_001.pdf]

Supporting Information to

Physiological stress response to sulfide exposure of freshwater anaerobic methanotrophic archaea

Maider J. Echeveste Medrano[1], Sarah Lee[1], Rob de Graaf[1], B. Conall Holohan[1], Irene Sánchez-Andrea[2,3], Mike S. M. Jetten[1], and Cornelia U. Welte*[1]

[1] Department of Microbiology, Radboud Institute for Biological and Environmental Sciences (RIBES), Radboud University, Heyendaalseweg 135, 6525AJ Nijmegen, The Netherlands.

[2] Department of Environmental Sciences for Sustainability, IE University, C. Cardenal Zúñiga 12, 40003 Segovia, Castilla-Leon, Spain

[3] Laboratory of Microbiology, Wageningen University, Stippeneng 4, 6708WE Wageningen, The Netherlands.

*Address correspondence to Cornelia U. Welte c.welte@science.ru.nl

Summary: 9 pages, 3 Figures, 7 Tables (Table 1-3 as separate excel table with three tabs)

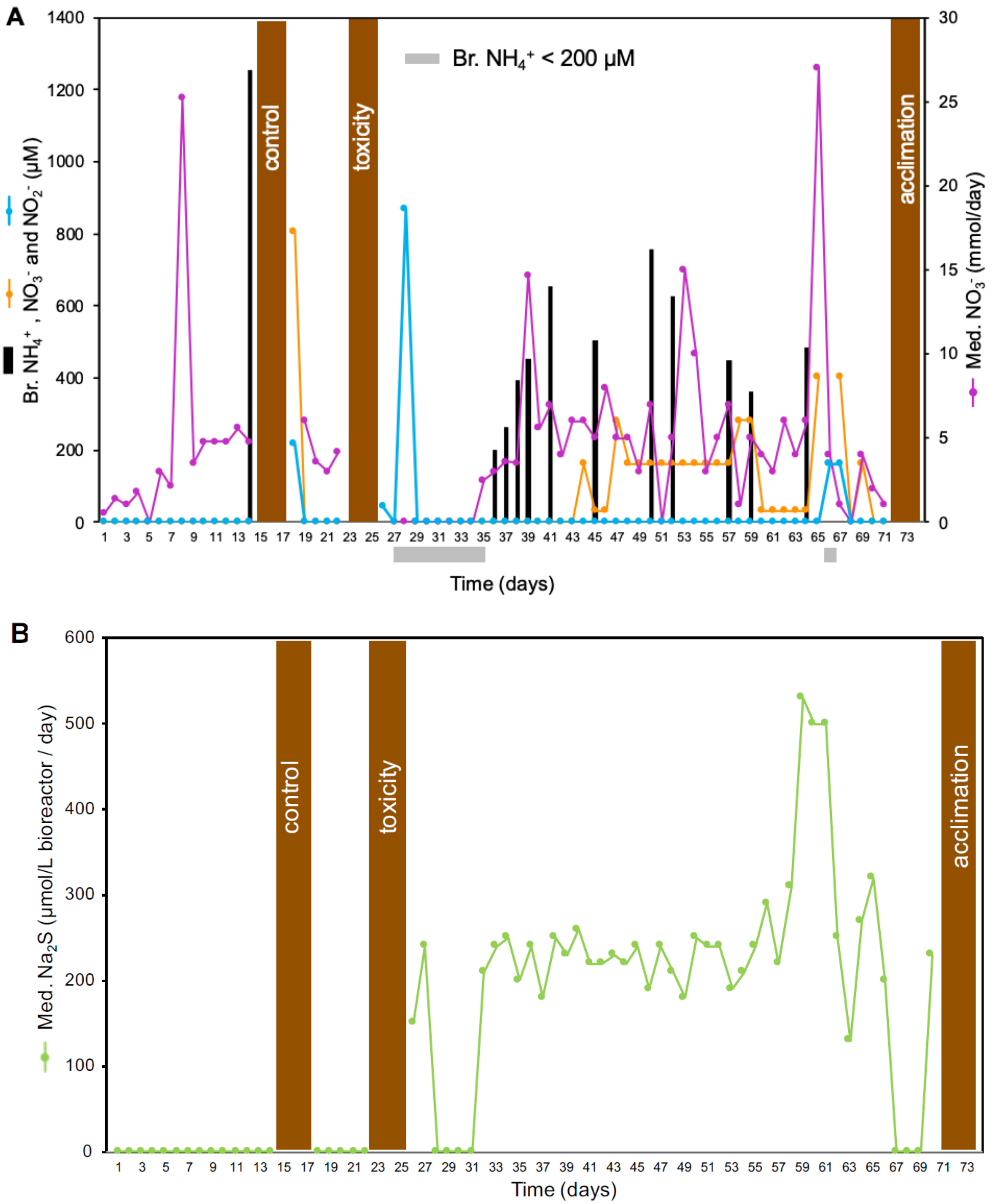

Supplementary Figure 1. A Medium feeding (Med.) and bioreactor *in situ* measured (Br.) liquid nitrogen (NH_4^+, NO_3^-, NO_2^-). At 18, 28 and 66 days, nitrite accumulation indicated a stressed microbial community and to prevent loss of the culture, nitrate feeding was reduced. Nitrate

accumulation on day 18 and days 43-69 indicated a reduced capacity of the microbial community to reduce nitrate. **B** Calculated sodium sulfide (Na_2S) addition over the sulfide experiment monitoring period (73 days) (x-axis). Sulfide was undetectable in the bioreactor medium at all time points. Vertically placed rectangular brown panels indicate whole bioreactor methane oxidation activity assays with ^{13}C-CH_4. Ammonium (NH_4^+) was measured once before the control activity assay and during the sulfide acclimation period. Ammonium with concentrations below 200 μM are indicated in horizontal panes in grey.

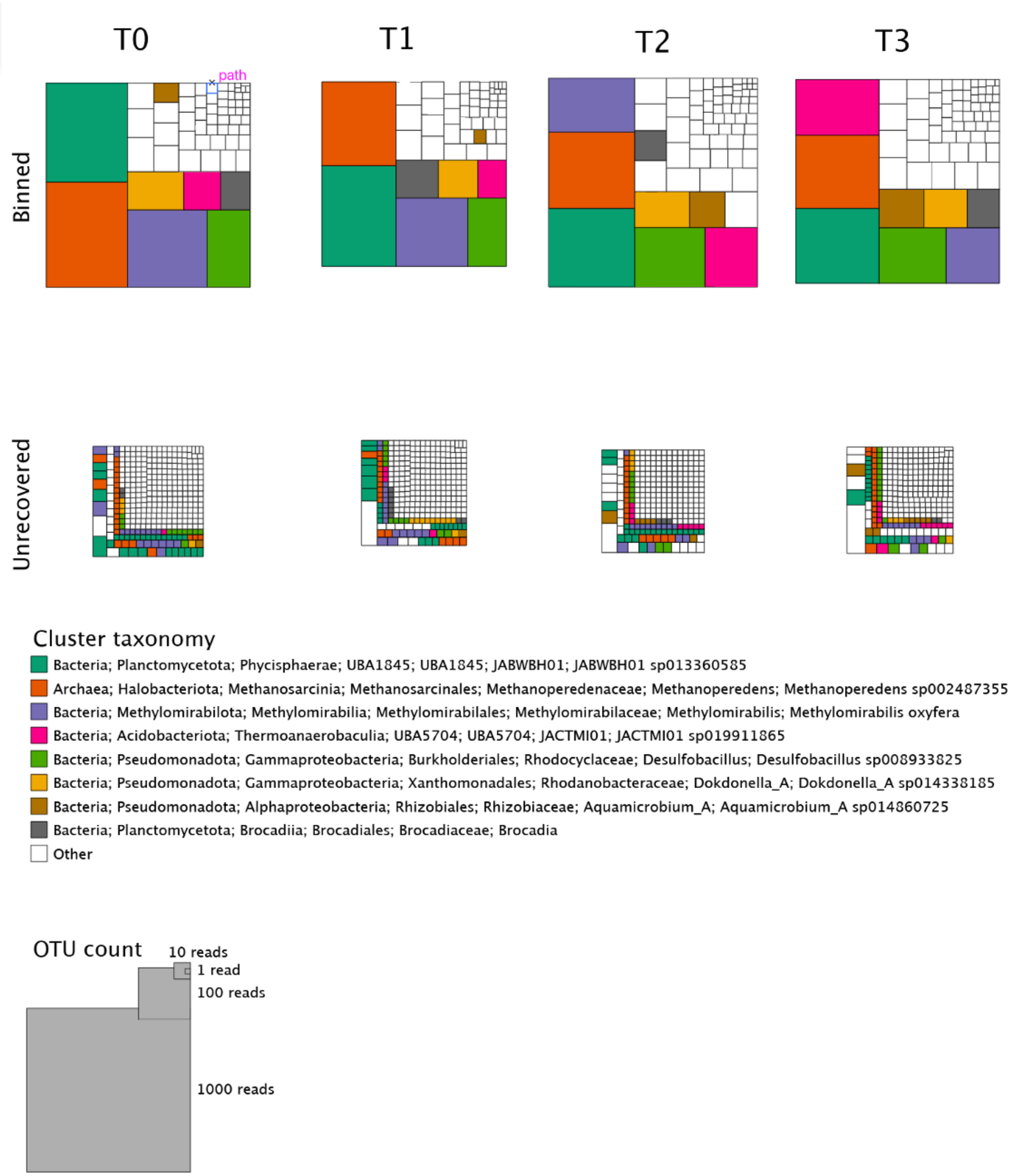

Supplementary Figure 2. SingleM read-based metagenome analysis quality based on recovery of taxonomical marker S3.1 ribosomal protein L2 rplB in the unbinned and binned fraction of the most abundant microorganism under the different conditions: T0, T1, T2 and T3

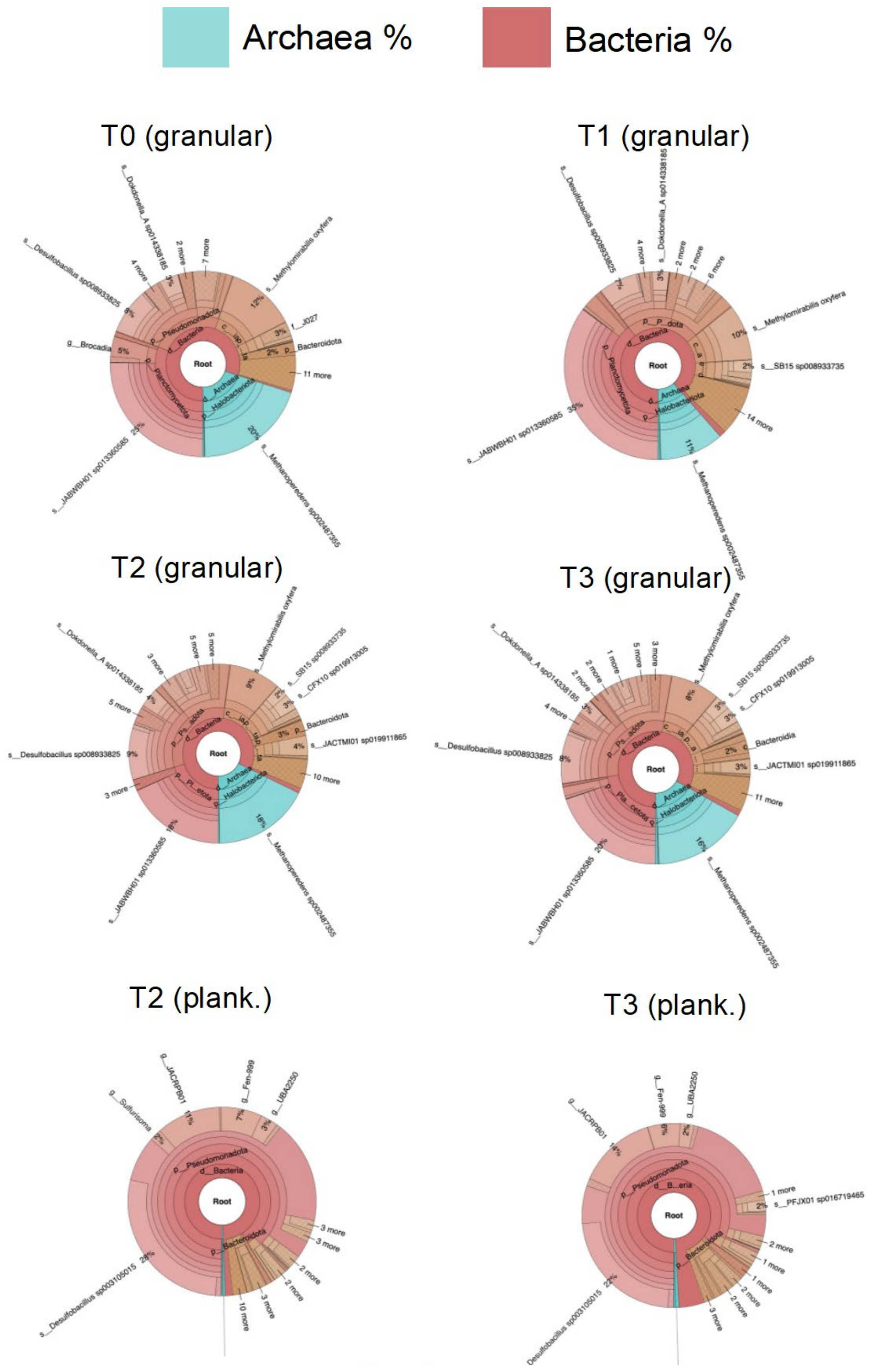
Archaea %
Bacteria %
T0 (granular)
T1 (granular)
T2 (granular)
T3 (granular)
T2 (plank.)
T3 (plank.)

Supplementary Figure 3. Results from the study on enriched biomass of the '*Ca.* Methanoperedens' morphotype. Taxonomical classification of the low-resolution metagenome, using SingleM, is shown as Krona diagrams (in %), comparing the granular fraction (all time points) and the planktonic fraction (T2 and T3), with emphasis on the archaeal ('*Ca.* Methanoperedens') and bacterial percentages.

Supplementary Tables 1-3 are included as a single excel file

Supplementary Table 4. The sulfide detoxifying/oxidizing contiguous cytochrome subunit of sulfide dehydrogenase (encoded by *fccA)* and sulfide dehydrogenase [flavocytochrome c] flavoprotein chain (encoded by *fccB)* [EC:1.8.2.3] gene transcript expression across all conditions in MAGs that showed a significant change of Padj<0.05 for at least condition (separately). Ordered from highest to lowest expression from T0-T1 condition. Condition differences (T0-T1, T0-T2 and T0-T3) indicate log2 fold chain (FC) values. "NA" (Not Available).

MAG	Gene (contig)	T0-T1	T0-T2	T0-T3
Thiobacillaceae_g_PFJX01	*fccA (17468_4)*	NA	-1.16	**-7.30**
Thiobacillaceae_g_PFJX01	*fccB (17468_3)*	NA	-2.68	**-5.83**
Thiobacillaceae_g_PFJX01	*fccA (1829_24)*	NA	-4.46	**-5.43**
Rhodocyclaceae_g_JACRPB01	*fccA (395_113)*	NA	**-6.26**	**-4.99**
Rhodocyclaceae_g_JACRPB01	*fccB (395_114)*	NA	**-8.12**	**-4.93**
Rhodocyclaceae_g_JACRPB01	*fccA (496_32)*	4.43	**-5.04**	**-5.81**
Desulfobacillus_2	*fccA (469_103)*	**3.28**	-0.08	**-3.77**
Thiobacillaceae_g_PFJX01	*fccB (3795_16)*	2.38	-4.66	**-5.57**
Rubrivivax	*fccA (183_163)*	2.05	0.00	**-3.59**
Rubrivivax	*fccB (183_162)*	0.97	-0.15	**-2.74**
Desulfobacillus	*fccA (1892_4)*	0.65	**-2.00**	-1.82
Desulfobacillus	*fccB (1892_6)*	0.46	-0.86	**-3.16**
Rubrivivax	*fccB (1138_43)*	-0.84	**-2.20**	-1.67
Casimicrobiaceae_g_JACPUX01	*fccB (261_94)*	-1.05	0.91	**3.56**
Thiobacillaceae_g_PFJX01	*fccB (1829_25)*	-2.46	**-3.88**	**-3.39**
Casimicrobiaceae_g_JACPUX01	*fccA (117_39)*	-3.01	1.54	**6.08**

Supplementary Table 5. Additional sulfur cycling gene transcript expression across all conditions in MAGs affiliated with methane oxidizers that showed a significant change of Padj<0.05 for at least one condition. Sulfate adenylyltransferase (sat) (KEGG: K00958) and L-cysteine S-thiosulfotransferase (soxX) (KEGG: K17223). MAGs have been ordered from highest to lowest level of expression in time point T0-T3. We only included gene expression shift with conditions were Padj <0.05. Condition differences (T0-T1, T0-T2 and T0-T3) indicate log2 fold chain (FC) values.

**MAG**	**Gene (contig)**	**T0-T1**	**T0-T2**	**T0-T3**
Ca. Methylomirabilis oxyfera	*sat (583_24)*	0.28	**3.72**	**4.70**
Ca. Methylomirabilis oxyfera	*soxX (34_5)*	0.56	**3.80**	**4.26**
Ca. Methylomirabilis oxyfera	*sat (767_16)*	-0.15	**1.30**	**2.17**
Ca. Methanoperedens BLZ2	*sat (836_68)*	**-1.03**	**1.27**	**1.90**

Supplementary Table 6. Transcriptional response of '*Ca.* Methanoperedens' PHA cycling marker genes. Poly[(R)-3-hydroxyalkanoate] polymerase subunit (PhaC) [EC:2.3.1.304], poly[(R)-3-hydroxyalkanoate] polymerase subunit (PhaE), acetyl-CoA C-acetyltransferase [EC:2.3.1.9] (AtoB) and 3-oxoacyl-[acyl-carrier protein] reductase [EC:1.1.1.100] (FabG). Padj<0.05 is indicated bold. Identifier in parenthesis refers to contig followed with an underscore for the Open Reading Frame (ORF).

**Gene** (contig)	**T0-T1**	**T0-T2**	**T0-T3**
PhaE (174_112)	**0.83**	-0.38	-0.33
PhaE (75_267)	**1.06**	**-0.81**	**-1.47**
PhaE (75_268)	0.69	0.43	0.48
PhaC (75_269)	0.53	0.66	-0.15
FabG (75_270)	0.13	-0.10	-0.96

AtoB (75_271)	1.02	0.00	-0.74
FabG (75_272)	0.48	0.11	0.35
PhaE (9357_4)	**0.76**	0.43	-0.36
PhaE (9357_5)	0.49	0.29	-0.12
PhaC (9357_6)	0.29	**0.88**	**0.43**
FabG (9357_7)	-0.07	**0.64**	**0.59**
AtoB (9357_8)	0.05	**0.91**	0.16
FabG (9357_9)	0.23	**0.78**	0.32
FabG (724_27)	**1.05**	0.23	-0.03
AtoB (724_28)	**0.86**	0.03	**-0.84**
FabG (724_29)	0.43	0.24	-0.07
PhaC (724_30)	**0.80**	0.08	**-1.06**
PhaE (724_31)	**-0.83**	0.08	0.81
PhaE (724_32)	0.01	**-0.96**	**-1.73**

Supplementary Table 7. Transcriptional response of '*Ca.* Methanoperedens' morphotype shift gene markers**.** Cell division genes (*ftsZ*), archaeal type IV pilus assembly (*pilA*), archaeal flagellin (*flaB*), archaeal flagellar protein genes (*flaF*/*flaH*/*flaI*/*flaJ*), chemotaxis protein methyltransferase genes *cheR*/*cheD*. Padj<0.05 is indicated in bold. Identifier in parenthesis refers to contig followed with an underscore for the Open Reading Frame (ORF).

**Gene (contig)**	**T0-T1**	**T0-T2**	**T0-T3**
FtsZ (174_152)	**0.53**	-0.27	**-0.66**
FtsZ (2651_3)	**0.71**	-0.21	**-0.65**
FtsZ (300_22)	**0.90**	**-0.38**	**-1.13**
FtsZ (300_166)	-0.09	0.34	**0.71**
FtsZ (447_69)	0.56	-0.21	0.22
FtsZ (724_84)	**0.80**	0.10	0.45
FtsZ (75_71)	1.12	-0.02	0.42
FtsZ (983_26)	**0.50**	-0.07	**-0.49**
PilA (3533_20)	**1.83**	1.15	0.58
PilA (75_204)	0.03	-1.35	0.35
FlaB (765_42)	1.18	1.06	1.65

FlaB (765_43)	**0.97**	0.20	-0.29
FlaB (765_44)	0.67	0.04	0.11
FlaF (765_45)	0.27	-0.46	-1.29
FlaG (765_46)	-2.23	1.00	1.67
FlaH (765_47)	-0.68	-1.17	0.10
FlaI (765_48)	-0.54	0.08	-0.20
FlaJ (765_49)	-0.03	0.06	-0.11
CheR (765_54)	-2.48	0.52	3.77
CheC (765_55)	-0.25	1.62	0.35
CheD (765_56)	-0.09	0.26	-0.93
FlaJ (174_127)	0.56	0.12	0.04
FlaI (174_128)	-1.14	0.26	1.33
FlaI (2294_11)	-0.74	-0.05	-0.98
FlaJ (2294_12)	0.76	-0.68	0.15
